# Editorial: Hormonal responses in women across the lifespan and their impact on physical performance

**DOI:** 10.3389/fendo.2026.2023902

**Published:** 2026-10-30

**Authors:** Eduardo Piedrafita, César Berzosa, Marta Sofía Valero, Ana Vanessa Bataller-Cervero

**Affiliations:** 1 Faculty of Health Sciences, Universidad San Jorge, Villanueva de Gállego, Spain; 2 Instituto Agroalimentario de Aragón-IA2, CITA-Universidad de Zaragoza, Zaragoza, Spain; 3 Departamento de Farmacología, Fisiología y Medicina Legal y Forense, Universidad de Zaragoza, Zaragoza, Spain

**Keywords:** exercise, female endocrinology, hormonal contraception, menopause, menstrual cycle, physical performance, polycystic ovary syndrome, women’s health

Hormonal variability is a defining feature of female physiology across the lifespan. From menstrual cycle fluctuations and hormonal contraception to endocrine disorders, the menopausal transition, and postmenopausal aging, changing hormonal environments interact with metabolic, neuromuscular, cardiovascular, inflammatory, psychological, and musculoskeletal systems. However, women remain underrepresented in exercise and sports science, while female-specific findings are sometimes translated prematurely into generalized recommendations. This Research Topic brings together 13 contributions that demonstrate both the biological relevance of hormonal status and the methodological caution required when interpreting its implications for health, exercise, and physical performance.

Several contributions address the menstrual cycle and athletic performance. Wen et al. synthesized evidence concerning aerobic, anaerobic, strength, and power outcomes across menstrual cycle phases, highlighting the inconsistency of reported effects, which is partly attributable to small sample sizes and inadequate phase verification. Zou et al. extended this perspective to musculoskeletal injury susceptibility. Although estradiol, progesterone, and relaxin provide plausible pathways linking hormonal variation to connective tissue properties, neural excitability, and neuromuscular control, the available evidence does not identify either a universally advantageous performance phase or a consistently high-risk injury window. Menstrual-cycle information should therefore complement, rather than replace, the individualized monitoring of symptoms, readiness, recovery, training load, and performance.

The distinction between biological plausibility and demonstrated functional relevance is also central to the critical review by Baber et al. Biomarkers of muscle damage and inflammation may vary among studies, but current evidence does not establish a reproducible phase-dependent pattern in post-exercise responses. Similarly, Yoshida et al. observed higher oxidative stress during the menstrual and follicular phases, particularly among participants with low iron stores, whereas hepcidin showed no consistent cyclical variation. Together, these studies underscore that biomarker interpretation depends not only on reproductive hormones, but also on iron status, the inflammatory context, biomarker kinetics, the exercise stimulus, and sampling time.

Beyond physiological measurement, endocrine health in sport requires appropriate knowledge and communication. Fischer et al. developed the Hormonal Contraception Knowledge Questionnaire, a brief instrument designed to identify knowledge gaps among female athletes and coaches. Its initial psychometric properties support further application and refinement, although its moderate internal consistency and the need for language-specific validation warrant caution. Importantly, this contribution shifts the focus from merely documenting hormonal exposure to supporting informed, athlete-centered conversations with qualified healthcare professionals.

Three studies examined polycystic ovary syndrome (PCOS), illustrating the need for integrated endocrine, metabolic, and psychosocial management. Atmaca et al. found no significant overall effect of pooled exercise interventions on total testosterone, dehydroepiandrosterone sulfate, or sex hormone-binding globulin, although aerobic exercise reduced total testosterone in modality-specific analyses. Zhao et al., by contrast, found no clear superiority of high-intensity interval training over moderate-intensity continuous training across anthropometric, cardiorespiratory, metabolic, or hormonal outcomes. These findings are complementary rather than contradictory: an active intervention may benefit women relative to control conditions without one exercise modality clearly outperforming another. Exercise prescription should therefore consider feasibility, preferences, clinical phenotype, supervision, and adherence.

Manickam and Pillai broadened the PCOS framework by highlighting psychological distress, altered self-perception, and trauma-related vulnerability. Although the small convenience sample, self-reported diagnoses, and cross-sectional design constrain generalizability and causal inference, the study reinforces the importance of compassionate, multidisciplinary, and appropriately trauma-informed care. PCOS should not be reduced to reproductive or metabolic abnormalities, nor should exercise and lifestyle recommendations be separated from the psychological context.

The menopausal transition emerges across this collection as a major period of systemic reorganization. Zhan et al. identified stage-associated lipidomic and leukocyte transcriptomic patterns, with particularly marked metabolic and inflammatory differences during the transition. These exploratory findings position this stage as a potential window for risk identification, while requiring longitudinal validation to separate endocrine effects from chronological aging. Luo et al. further conceptualized menopause as multisystem endocrine remodeling involving immune regulation, vascular function, brain metabolism, bone turnover, and tumor microenvironments. This systems-level perspective emphasizes that consequences relevant to physical function may arise through interacting biological pathways rather than isolated hormonal effects.

At the clinical level, Wang et al. reported that both overall and central adiposity were associated with menopause-related symptom severity, but estradiol statistically mediated only the association involving waist-to-hip ratio. Guo et al. similarly found that sleep quality statistically accounted for part of the association between menopausal and depressive symptoms. Because both studies were cross-sectional, their mediation estimates should be interpreted as indirect statistical associations rather than causal pathways. Nevertheless, they highlight potentially modifiable domains, including central adiposity and sleep, which deserve further prospective and interventional investigation.

Finally, Xu et al. quantified the substantial global burden of low back pain among women aged 55 years and over, which was used as a proxy for postmenopausal status. Absolute cases and disability increased markedly between 1990 and 2021, despite modest declines in age-standardized rates. High body mass index and occupational or environmental exposures represented key modifiable factors, emphasizing that hormonal aging operates within broader demographic, metabolic, and socioeconomic contexts. Maintaining physical activity, musculoskeletal capacity, and healthy body composition may therefore have implications extending well beyond competitive performance.

Collectively, the contributions to this Research Topic argue against hormonal determinism. Hormonal transitions matter, but their consequences are shaped by symptoms, clinical conditions, body composition, nutritional and iron status, exercise modality, psychosocial context, aging, and methodological choices. Future research should prioritize prospective designs, rigorous reproductive-stage classification, repeated hormone measurements, standardized exercise protocols, clinically meaningful outcomes, diverse populations, and sufficiently powered within-person analyses. Translating this evidence into practice requires flexible, individualized, and interdisciplinary approaches that support women’s health and physical function without imposing unsupported phase-based rules. Such approaches should integrate objective physiological measures with women’s reported experiences, recognize variation both within and between individuals, and distinguish population-level associations from recommendations for a particular athlete or patient.

Across the collection, a priority is methodological transparency. Studies should report cycle characteristics, hormonal verification procedures, contraceptive formulations, menopausal staging, symptom burden, medication use, training status, and comorbidities with sufficient precision. Harmonized reporting would improve comparability while preserving biologically meaningful heterogeneity. Co-designed research involving athletes, patients, coaches, clinicians, and exercise professionals may strengthen outcome selection, implementation, and translation. Ultimately, better evidence will depend not simply on including more women, but on studying female physiology with appropriate designs, measurements, and interpretations.

